# Seasonality, epidemiology and phylogeny of *Theileria ovis* with a note on hematological and biochemical changes in asymptomatic infected goats from Pakistan

**DOI:** 10.1371/journal.pone.0290620

**Published:** 2023-08-29

**Authors:** Muhammad Irfan, Shun-Chung Chang, Rana Khalid Iqbal, Muhammad Tanveer, Muhammad Asif, Adil Khan, Nasreen Nasreen, Farhan Ahmad Atif, Rehan Sadiq Shaikh, Munir Aktas, Mourad Ben Said, Furhan Iqbal, Chien-Chin Chen

**Affiliations:** 1 Institute of Molecular Biology and Biotechnology, Bahauddin Zakariya University Multan, Multan, Pakistan; 2 Department of Emergency Medicine, Ditmanson Medical Foundation Chia-Yi Christian Hospital, Chiayi, Taiwan; 3 Institute of Zoology, Bahauddin Zakariya University Multan, Multan, Pakistan; 4 Department of Botany and Zoology, Bacha Khan University, Charsadda, Khyber Pakhtunkhwa, Pakistan; 5 Department of Zoology, Abdul Wali Khan University Mardan, Khyber Pakhtunkhwa, Pakistan; 6 Medicine Section, Department of Clinical Sciences, College of Veterinary and Animal Sciences, Jhang, Sub-campus of University of Veterinary and Animal Sciences, Lahore, Pakistan; 7 Center of Applied Molecular Biology, Thokar Niaz Baig Lahore, Pakistan; 8 Department of Parasitology, Veterinary Faculty, University of Firat, Elazig, Turkey; 9 Department of Basic Sciences, Higher Institute of Biotechnology of Sidi Thabet, University of Manouba, Manouba, Tunisia; 10 Laboratory of Microbiology, National School of Veterinary Medicine of Sidi Thabet, University of Manouba, Manouba, Tunisia; 11 Department of Biotechnology and Bioindustry Sciences, College of Bioscience and Biotechnology, National Cheng Kung University, Taina, Taiwan; 12 Department of Pathology, Ditmanson Medical Foundation Chia-Yi Christian Hospital, Chiayi, Taiwan; 13 Department of Cosmetic Science, Chia Nan University of Pharmacy and Science, Tainan, Taiwan; 14 Ph.D. Program in Translational Medicine, Rong Hsing Research Center for Translational Medicine, National Chung Hsing University, Taichung, Taiwan; King Abdulaziz City for Science and Technology (KACST), SAUDI ARABIA

## Abstract

Caprine theileriosis, caused by *Theileria ovis* is a serious production issue, especially in the areas that depend on goats and sheep for milk, meat, and other economic benefits. Pakistan has a large goat population, but few reports have been documented from this country regarding PCR-based detection of *T*. *ovis*. The molecular prevalence of *T*. *ovis*, on a seasonal basis, in various goat breeds enrolled from Muzaffar Garh district of Punjab in Pakistan was determined from October 2018 to September 2019. In this study, 1084 goat blood samples were screened for the detection of *T*. *ovis* DNA through PCR-based amplification of 18S rRNA gene. Out of 1084 goats, 12 (1.11%) were infected with *T*. *ovis*. The parasite prevalence varied with the sampling seasons (Chi square test, P = 0.008), and the parasite prevalence was highest in goat blood samples collected in summer (2.39%) followed by winter (1.88%). DNA sequencing and BLAST analysis confirmed the presence of *T*. *ovis*, and the amplified isolates from the 18S rRNA gene of *T*. *ovis* were found to be highly conserved during phylogenetic analysis. Young goats (Fischer exact test, P = 0.022) were found more infected with *T*. *ovis* during the winter season. Infected goats had elevated white blood cell counts (Two-sample t-test, P = 0.04), blood urea nitrogen to Creatinine ratio (Two-sample t-test, P = 0.02) and decreased serum Creatinine (Two-sample t-test, P = 0.001) as compared to *T*. *ovis* negative goats. We report a relatively low molecular prevalence of *T*. *ovis* in goats from the Muzaffar Garh district. However, it is recommended that control measures to eradicate *T*. *ovis* infection in goats in this area should be taken.

## Introduction

In Pakistan’s agricultural economy, livestock is the backbone of the animal production industry, accounting for 11.8% of the overall GDP and contributing around $801.3 billion in revenue [1]. Pakistan is the third biggest goat-producing nation, with approximately 60 million heads divided into about 37 breeds reported from various parts of the country [2]. During the financial year 2019–2020, small ruminants contributed 1 million tons of milk, 0.75 million tons of meat, 0.47 million tons of wool, and 0.29 million tons of hair to the national economy of Pakistan [3].

Despite Pakistan’s large goat population, the output of these animals is not as high as it should be. This disparity is attributed to various factors, including the selection of poor breeders, deficits in management, feed availability, and the incidence of vector-borne diseases [4]. However, due to its subtropical climatic conditions, Pakistan provides ideal conditions for the growth and reproduction of ticks, which are the most common vectors of several livestock diseases in Pakistan, resulting in substantial economic losses [5].

Ovine and Caprine theileriosis is a tick-borne parasitic disease that causes significant economic losses in small ruminants [6, 7]. Economic losses are mainly due to high mortality, lower productivity, higher costs of treating sick animals, and vector control [8]. Several protozoan species belonging to the genus *Theileria* (*T*. *ovis*, *T*. *lestoquardi*, *T*. *separata*, and newly identified species: *Theileria* sp.-China 1) are responsible for theileriosis in sheep and goats with variable virulence [9, 10]. Fever, enlarged lymph nodes, anorexia, weakness, anemia, and cough are common symptoms of this pathological condition. Some animals were also reported to have pyrexia, leucopenia, and pale mucous membranes [7]. If infected animals are not treated, they can die within 3–4 weeks [6, 11].

Previously, microscopic analysis of blood smears (Giemsa-stained) and clinical symptom evaluation were considered the standard method for theileriosis diagnosis [7]. This method is no longer standard, given that microscopic examination is useful only in cases where animals have high parasitemia and are suffering from acute infection [12]. However, it is now established that polymerase chain reaction (PCR) based amplification is a superior tool to microscopic inspection for identifying those small ruminants that are infected with theileriosis (carriers), but they have not yet developed the disease signs [13, 14].

Despite a large goat population, there are very few reports from Pakistan in which PCR-based detection of *T*. *ovis* has been documented from Pakistan [14–17]. Interestingly, the effect of season on *T*. *ovis* prevalence in goats has never been reported from any part of this country. The present study aimed to report the molecular prevalence of *T*. *ovis* in the blood samples of Pakistani goats on a seasonal basis and to report the associated risk factors. Phylogenetic positioning of revealed *T*. *ovis* isolates was also performed based on the analysis of the 18S rRNA partial gene sequence.

## Materials and methods

### Study area

**Fig 1:**
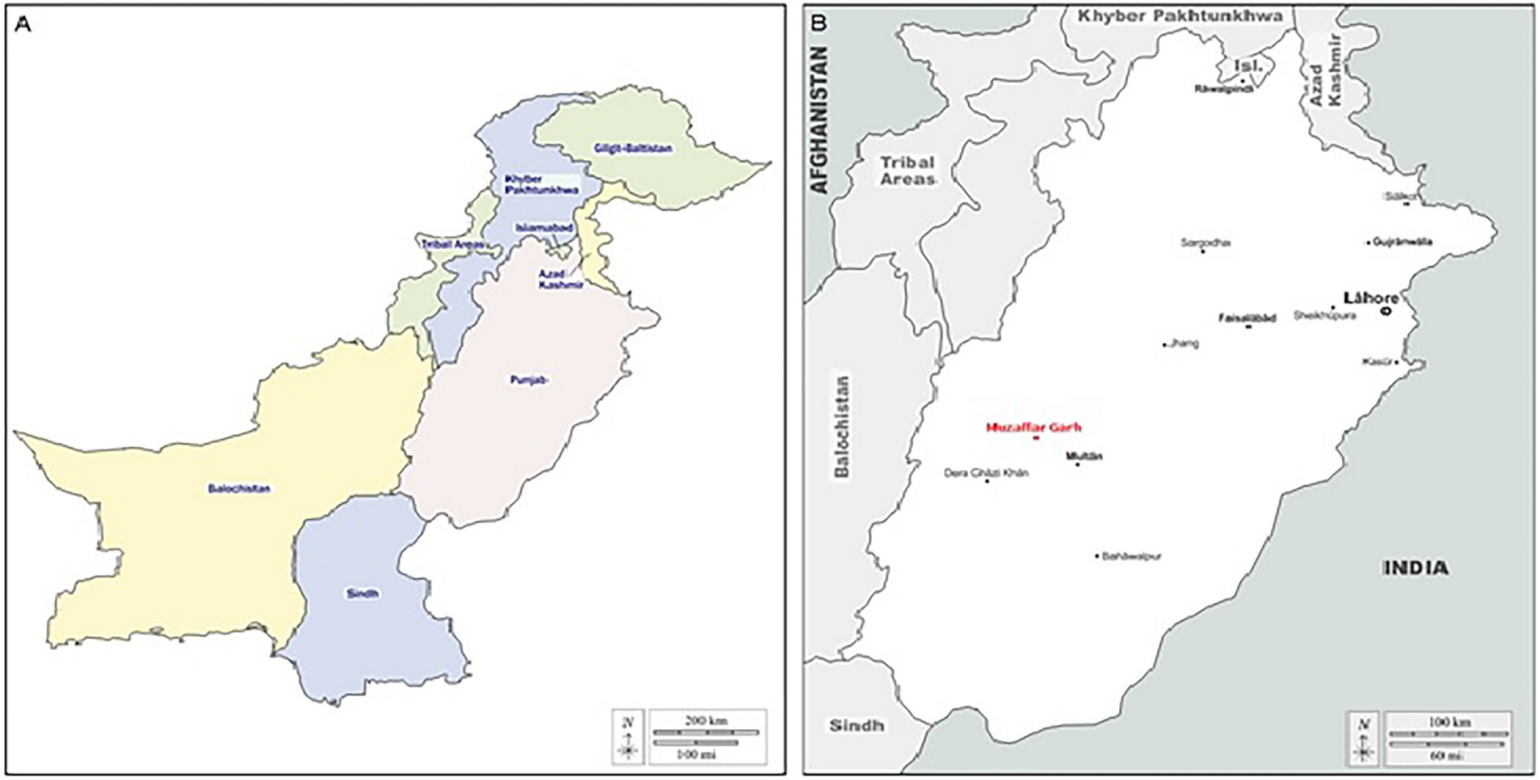
Maps of Pakistan showing Punjab Province (A) and Punjab province showing Muzaffar Garh district (B).

Muzaffar Garh is one of the oldest districts in Punjab, with an area of 8,249 Km^2^. The district is flanked to the north by Layyah, Rahimyar Khan, and Bahawalpur to the south and by Khanewal and Multan in the east, while Jhang district has a northeast boundary with it. Dera Ghazi Khan and Rajanpur districts are located on the western side [18] (Fig 1). Muzaffar Garh has an arid environment with a sweltering summer and a moderate winter. The highest temperature ever recorded in this area was 54°C (129°F) while 1°C (30°F) was the lowest temperature that was recorded. The average amount of rainfall is 127 mm (5.0 in) [18].

### Experimental design

According to a recent estimate by Malik et al. [18], Muzaffar Garh district has around 0.508 million goat heads, and in order to calculate the goat number to be enrolled during the present investigation, Solvin’s formula was applied following Zafar et al. [19]. The estimated sample size was 1000 goats. A total of 1084 healthy goats were randomly selected from various herds in the Muzaffar Garh district of Punjab (Pakistan) from October 2018 to September 2019, following the consent from livestock owners. Enrolled goats belonged to nine different breeds [Teddy (N = 32), Lail Puri ((N = 17), Sindhi (N = 21), Daira Din Pannah (N = 806), Dessi (N = 6), Roucher (N = 4), Nukri (N = 102), Makhi Cheena (N = 65) and Beetal (N = 31)]. None of the analyzed goats were blood sampled more than once during the present investigation, and during each season, different goats were enrolled in the study and tested. May till September was defined as summer, October till November was autumn, December till January was winter, and February till April was the spring. For each enrolled goat at a particular sampling site, a questionnaire was conducted to gather data about the herd and the animal to estimate the risk factors potentially associated with the prevalence of *T*. *ovis*. Ethical Research Committee of the Institute of Molecular Biology and Biotechnology at Bahauddin Zakariya University Multan (Pakistan) approved all the experimental procedures and protocols applied in this study via letter number IMBB/Ethics/2019-24 and all the experiments were performed in accordance with the relevant guidelines and regulations of Bahauddin Zakariya University Multan (Pakistan) following the Helsinki declaration. Data reported in this manuscript was generated follows the recommendations in the ARRIVE guidelines.

### Tick collection and identification

The whole body of each goat was thoroughly investigated for the presence of ticks. When ticks were observed, mouth-blunted forceps were used to collect them from the goats carefully. Ticks were transferred to 70% ethanol-containing Eppendorf tubes, and later on, they were morphologically identified under a stereo-zoom microscope by following morpho-taxonomic characters of ticks according to the relevant standard identification key [20].

### Blood sampling and hematological, biochemical, and enzyme activity analysis

The jugular vein of each goat was aseptically punctured to sample 5 ml of blood. About 3ml was collected in a labeled tube that contained 0.5 M EDTA. These samples’ complete blood count (CBC) was determined using a hematology analyzer (Sysmex KX21, Japan). The residual blood after CBC analysis was used later on to extract DNA. The remaining blood that was collected from each animal, and was not added with EDTA, was centrifuged at high speed for at least 10 minutes to separate the serum that was stored at -20°C until it was analyzed to determine the Aspartate aminotransferase, creatinine, Alanine aminotransferase, blood urea nitrogen, urea, and blood urea nitrogen to creatinine levels by using a biochemistry analyzer (SelectraProM, Australia).

### DNA isolation and PCR amplification

The inorganic approach was used to extract DNA from blood samples, as described by Saeed et al. [21]. A pair of primers (forward 5’- TCGAGACCTTCGGGTGGCGT-3’and reverse 5’-TCCGGACATTGTAAAACAAA-3’) was applied to amplify a 520 bp fragment from the 18S rRNA gene of *T*. *ovis* according to Altay et al. [22]. A 50 μl reaction volume was prepared to perform PCR. The reaction mixture contained 10X buffer [composed of 100 mM Tris-HCl (pH 9.1) and 500 mM KCl], genomic DNA (approximately 250 ng), 0.16 mM of dNTPs, 1.5 mM MgCl_2,_ 12 pM of each primer and 2 U of Taq DNA polymerase (Vivantis, UK). Reaction conditions included an initial denaturation step for 5 minutes at 94°C followed by 30 cycles of denaturation for 30 seconds at 94°C, annealing for 45 seconds at 59°C, and elongation for 1 minute at 72°C. The last extension step was performed for 7 minutes at 72°C. The amplified PCR products were resolved on a 2% agarose gel, and a 100 bp DNA ladder (Vivantis, USA) was used to confirm the presence of 520 bp amplicon of 18S rRNA gene specific to *T*. *ovis*. *Theileria ovis* confirm positive DNA samples generated from sheep during our previous study (accession number MZ648443, MZ648444 and MZ648445) [10] was used as a positive control during each PCR. In addition, a PCR reaction mixture without template DNA was used as a negative control.

### Theileria ovis sequencing, sequence alignment, and phylogenetic analysis

**Fig 2:**
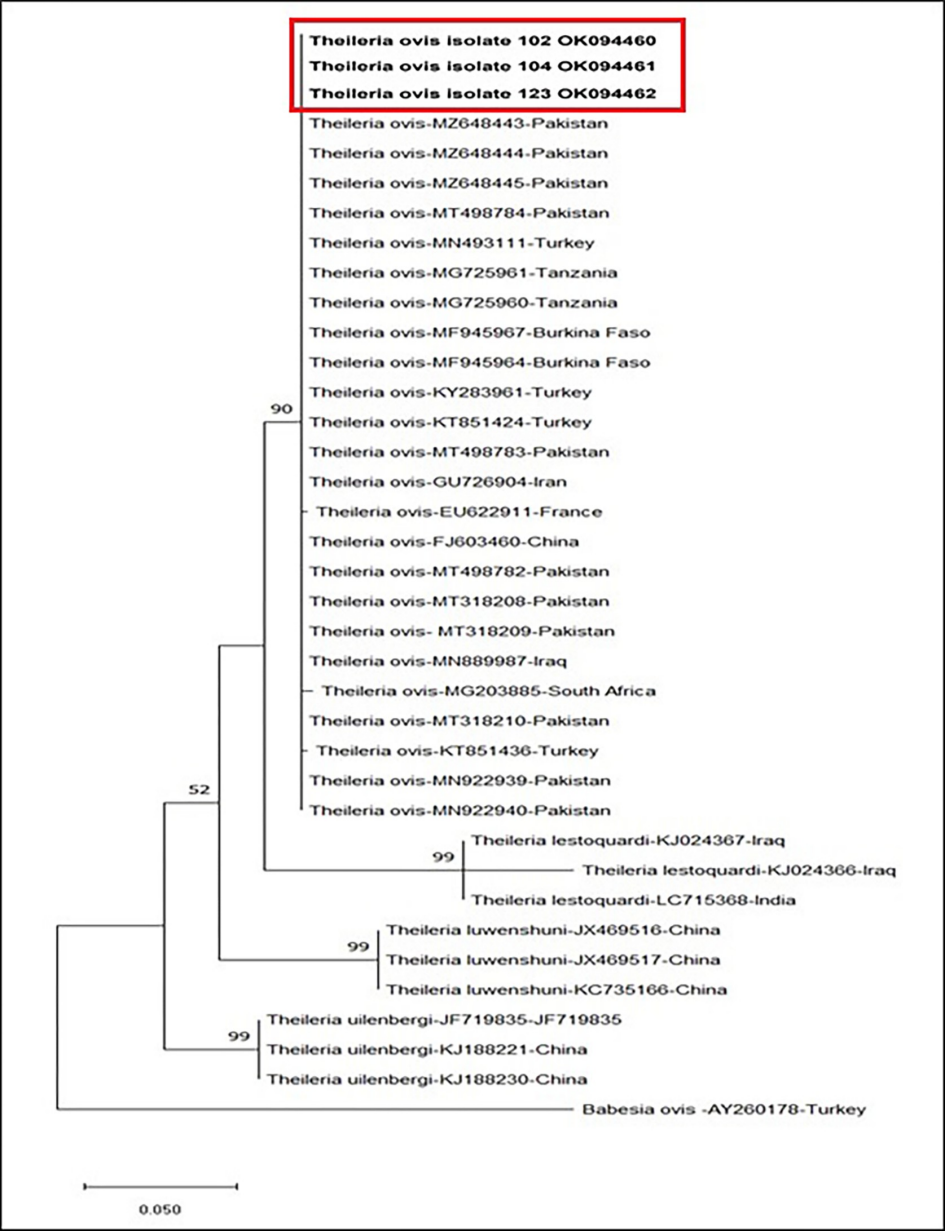
Phylogenetic tree based on a partial sequence (482 bp) of 18S rRNA gene of *Theileria ovis* isolated in this study (bold letters) with other small ruminants *Theileria* species in GenBank from around the world. The evolutionary history was inferred by using the Maximum Likelihood method and Kimura 2-parameter model [24]. The percentage of replicate trees in which the associated taxa clustered together in the bootstrap test (1000 replicates) is shown next to the branches. Only bootstrap values >50 is indicated next to branches. GenBank accession numbers are indicated on the right of each species name. *Babesia ovis* (AY260178) was used as an outgroup. The scale-bar represents the evolutionary distance in the units of the number of nucleotide substitutions per site. Evolutionary analyses were conducted in MEGA X [23].

PCR products of 18S rRNA partial sequence amplified from *T*. *ovis* DNA were sequenced by First base (Selangor, Malaysia). BLAST analysis confirmed the *T*. *ovis* infection, and the 18S rRNA gene sequences were deposited in GenBank. Phylogenetic analysis was conducted by aligning 482 nucleotides of the 18S rRNA gene and MEGA X was used to construct a phylogenetic tree [23]. The Maximum Likelihood approach and the Kimura 2-parameter model were applied to infer the evolutionary history [24]. The tree with the greatest log probability (-1400.96) was displayed (Fig 2). This analysis involved 37 nucleotide sequences.

### Statistical analysis

Minitab (Minitab, USA) was used for data analysis. Mean values ± standard error of the mean was the mode of data presentation. *P* ≤ 0.05 was set as the significance level. Fisher exact test was used to correlate *T*. *ovis* prevalence with the studied epidemiological factors. Hematobiochemical parameters were compared between animals positive and negative for *T*. *ovis* by applying the two-sample t-test. One-way ANOVA was applied to compare the *T*. *ovis* prevalence among the enrolled goat breeds. While a Chi-square test was conducted to compare *T*. *ovis* prevalence among the four sampling seasons.

## Results

### Morphological identification of ticks

As most of the enrolled goats were farmed, they were well maintained, and only a small number of ticks (N = 62) were collected from them upon identification. It was observed that the identified ticks were *Rhipicephalus sanguineus*, *Hyalomma anatolicum* and *Hyalomma marginatum*.

### Molecular prevalence and phylogenetic analysis of T. ovis

The overall prevalence rate of *T*. *ovis* was 1.11% (12/1084) during the present study (Table 1). A total of three *T*. *ovis* isolates were sequenced, and only one genotype was identified upon the alignment of partial 18S rRNA nucleotide sequences (482 bp). These three isolates, named 104, 102, and 123, were deposited in GenBank with accession numbers OK094460, OK094461, and OK094462, respectively. A 99–100% sequence homology of the DNA generated in this study was revealed by BLAST analysis to the 18S rRNA partial sequences of *T*. *ovis* sequences already available in GenBank (Fig 2). Phylogenetic analysis confirmed that our isolates were clustered with those previously reported from Pakistan (MN922939, MN922940, MT498784, MT318210, MZ648443, MZ648444, MZ648445, MT498782, MT318208, MT318209), Turkey (MN493111, KY283961, KT851436), Iran (GU697204), China (FJ603460), Burkina Faso (MF945964, MF945967), Iraq (MN889987), South Africa (MG203885) and Tanzania (MG725960, MG725961) (Fig 2).

**Table 1:** Overall and seasonal prevalence of *Theileria ovis* in blood samples of goats collected in this study from Muzaffar Garh district in Pakistan.

Sampling season	Number	*T*. *ovis* positive samples/total (%±C.I.^1^)	*P* value^2^
Autumn	262	0/262 (0)	0.008*
Winter	265	5/265 (1.88±0.01)	
Spring	265	0/265 (0)	
Summer	292	7/292 (2.39±0.01)	
Total	1084	12/1084 (1.11±0.01)	

Abbreviations

^1^: C.I.: 95% confidence interval.

^2^: *P*-value is calculated according to seasons.

*: Statistically significant test.

### Risk factors analysis

The prevalence of *T*. *ovis* in goats significantly varied with the sampling seasons (*P* = 0.008), and the prevalence of *T*. *ovis* was highest in goats tested in summer (2.4%), followed by those analyzed in winter (1.9%). The collected goat samples during spring and autumn were not infected with *T*. *ovis* (Table 1). When the prevalence of *T*. *ovis* was compared among the analyzed goats, it was observed that the highest infection rate was observed in the Lail Puri breed (20%) followed by Daira Din Panah (3.24%) and Makhi Cheena (2.78%) breeds while the tested Sindhi, Nukri, Beetal and Teddy breed goats were not found to be infected with *T*. *ovis*. During the winter season, Daira Din Pannah was the only goat breed that was infected with *T*. *ovis* (1.9%). One-way ANOVA results revealed that *T*. *ovis* infection between goat breeds did not vary with sampling seasons (*P* = 0.15 and 0.99, respectively, for summer and winter) (Table 2).

**Table 2:** Molecular prevalence results of *Theileria ovis* infection in analysed goats from Muzaffar Garh district during summer and winter seasons according to different risk factors.

Rick factors	Classes	*T*. *ovis* positive samples/total (infection rate (%) ± C.I.^1^)			
		Winter	*P*-value^2^	Summer	*P*-value^2^
Breed	Teddy	0/1 (0)	0.995	0/19 (0)	0.147
	Lail Puri	0/3 (0)		1/5 (20±0.35)	
	Sindhi	-		0/14 (0)	
	Daira Din Pannah	5/247 (2.02±0.01)		5/154 (3.24±0.02)	
	Dessi	0/3 (0)		-	
	Roucher	0/2 (0)		-	
	Nukri	-		0/49 (0)	
	Makhi Cheena	-		1/36 (2.78±0.05)	
	Beetal	-		0/15 (0)	
Gender	Male	1/30 (3.33±0.06)	0.537	0/46 (0)	0.247
	Female	4/235 (1.70±0.01)		7/246 (2.84±0.02)	
Age	≤ 24 months	5/131 (3.82±0.03)	0.022*	6/151 (3.97±0.03)	0.068
	> 24 months	0/134 (0)		1/141 (0.71±0.01)	
Water resource	Pump	0/39 (0)	0.349	0/24 (0%)	0.423
	Pool	5/226 (2.21±0.01)		7/268 (2.61±0.01)	
Tick load on goats	Yes	-	NC	7/253 (2.77±0.01)	0.293
	No	5/265 (1.89±0.01		0/39 (0)	
Other dairy animals at farm	Yes	5/200 (2.50±0.02)	0.198	7/271 (2.58±0.01)	0.456
	No	0/65 (0)		0/21 (0)	
Dogs at farm	Yes	5/265 (1.89±0.01)	NC	6/242 (2.48±0.01)	0.840
	No	-		1/50 (2±0.03)	
Tick load on dogs	Yes	-	NC	6/242 (2.48±0.01)	0.840
	No	5/265 (1.89±0.01)		1/50 (2±0.03)	
Number of goats in herd	≤ 20	-	NC	-	NC
	> 20	5/265 (1.89±0.01)		7/292 (2.40±0.01)	
Total		5/265 (1.89±0.01)		7/292 (2.40±0.01)	

Abbreviations

^1^: C.I.: 95% confidence interval

2: *P*-values indicate the results of Fisher exact test calculated for each studied factor in each season.

*: Statistically significant test.

NC = Not calculated.

Risk factor analysis indicated that the age of goats tested in the winter was the only parameter found associated with the prevalence of *T*. *ovis* (Fisher exact test, *P* = 0.022), and it was observed that young goats (up to 24 months old) were more susceptible to *T*. *ovis* infection. All other studied risk factors (gender, tick load on goats, dogs associated with the herd, tick load on dogs, presence of other dairy animals on a farm, drinking water resources) do not seem to be incriminated in the presence or absence of *T*. *ovis* infection of goats sampled during summer and autumn seasons (*P* > 0.05, Table 2).

### Hematological, biochemical, and enzyme activity analysis

It was observed that serum creatinine concentrations were substantially lower (two-sample t-test, *P* = 0.001) while blood urea nitrogen to creatinine ratio was significantly elevated (two-sample t-test, *P* = 0.02) in *T*. *ovis* infected goats than in uninfected goats during the winter season. Among complete blood count parameters, white blood cell count was significantly elevated in goats infected with *T*. *ovis* during winter compared to those not infected with this parasite (*P* = 0.04). All other studied hematochemical parameters varied non-significantly (*P* > 0.05) when compared between *T*. *ovis* infected and uninfected goats sampled during the summer and winter seasons (Table 3).

**Table 3:** Comparison of haematochemical parameters between *Theileria ovis* positive and negative goat blood samples collected from Muzaffar Garh district during summer and winter seasons.

Parameters	Winter			Summer		
	*T*. *ovis* positive (N = 05)	*T*. *ovis* negative (N = 260)	*P value*	*T*. *ovis* positive (N = 07)	*T*. *ovis* negative (N = 285)	*P value*
**Serum biochemical parameters**						
Aspartate amino transferase (U/L)	125.2±7.7	107.2±1.7	0.08	148.0±32	123.4±2.9	0.5
Urea (mg/dl)	37.0±2.9	42.2±0.99	0.2	44.9±6.1	49.7±0.88	0.5
Creatinine (mg/dl)	0.36±0.024	0.73±0.023	0.001***	0.74±0.013	0.75±0.02	0.9
Blood urea nitrogen (mg/dl)	17.24±1.3	19.58±0.46	0.2	21.04±2.8	23.17±0.40	0.5
Blood urea nitrogen to Creatinine ratio	48.9±5.1	29.3±0.85	0.02*	32.7±5.7	33.0±0.79	0.9
**Complete blood count parameters**						
WBC count	28.72±3.7	17.77±0.36	0.04*	15.60±2.0	16.4±0.74	0.7
Neutrophils (%)	30.0±6.6	31.9±1.0	0.8	15.6±7.7	19.8±1.3	0.6
Lymphocytes (%)	67.4±6.5	65.4±1.0	0.8	81.7±7.4	76.4±1.3	0.5
Monocytes (%)	1.60±0.24	1.55±0.06	0.9	1.42±0.37	1.63±0.06	0.6
Eosinophilis (%)	0.80±0.20	1.2±0.03	0.1	1.14±0.26	1.43±0.04	0.3
Red blood cells (x 10^6^μL^-1^)	2.19±0.22	2.53±0.04	0.2	2.51±0.74	2.35±0.18	0.8
Hemoglobin (gdL^-1^)	7.96±0.14	7.84±0.05	0.5	9.6±1.6	8.25±0.17	0.4
Hematocrit (%)	20.92±3.0	25.9±0.18	0.2	24.1±5.0	22.0±0.64	0.7
Mean cell volume (f L)	94.1±4.4	101.6±0.64	0.2	103.8±5.7	102.0±1.0	0.8
Mean cell hemoglobin (pg)	37.24±2.5	32.57±0.39	0.1	42.8±3.7	41.6±0.62	0.8
Mean corpuscular hemoglobin concentration (g/dl)	40.24±3.9	32.34±0.44	0.1	41.06±2.6	40.3±0.64	0.8

Note: Data is expressed as mean ± standard error of mean. P value indicates the result of two-sample t test calculated for each studied parameter.

*P* > 0.05 = Non significant; *P* < 0.05 = Least significant (*); *P* < 0.001 = Highly significant (***).

## Discussion

Theileriosis is a tick-borne parasitic infection responsible for substantial economic losses worldwide to the livestock industry, as morbidity and mortality rates associated with this disease are high [11]. *T*. *ovis* is reported to be extensively widespread in Asia, Africa, and Europe, and it is thought to cause subclinical infection in sheep and goats, as opposed to the virulent *T*. *lestoquardi* [6, 12, 22]. Ticks infesting the small ruminants have not been reported in great detail from Pakistan, but it has been documented that *Rhipicephalus sanguineus*, *Rhipicephalus microplus*, *Hyalomma anatolicum* and *Hyalomma marginatum* are among the common ticks that infest local sheep and goats [25, 26]. Our results also agree with the previous reports as *Rhipicephalus sanguineus*, *Hyalomma anatolicum* and *Hyalomma marginatum* were collected and identified from the goats enrolled during the present study.

In this study, a pioneer investigation was performed in the Muzaffar Garh district of Punjab, where the *T*. *ovis* prevalence has been documented seasonally in goats. This district is an important livestock production region where livestock rearing is central to the livelihoods of local people. In the present study, 1.12% of investigated goats were infected with *T*. *ovis* (Table 1).

Until now, few reports are available in the literature in which the prevalence of T. ovis has been reported in Pakistani goats using the molecular tool: PCR. Recently, Niaz et al. [27] collected 800 blood samples from apparently healthy small ruminants (goats and sheep) from Malakand, Bajaur, Swat, and Shangla districts in Khyber Pakhtunkhwa (KPK) Province and 14.3% of the tested animals were found to be infected with *T*. *ovis*. Khan et al. [11] have also reported that 30.7% of analyzed goats from the northern highlands of Balochistan were infected with *T*. *ovis*. Riaz et al. [17] found that 31.8% of investigated small ruminants from Multan District were positive for the presence of *T*. *ovis*. In 2013, Iqbal et al. [15] reported that 16% of the analyzed goat blood samples from two provinces, Punjab (from Multan District) and KPK (from Kohat District), were found positive for *T*. *ovis* by using Reverse line blot assay. In 2012, a relatively low prevalence (6%) of *T*. *ovis* was recorded by Durrani et al. [28] when they analyzed small ruminant blood samples collected from two provinces of Pakistan (Punjab and KPK). It was reported that *T*. *ovis* prevalence was higher in small ruminants of KPK than in Punjab, and Durrani et al. [28] proposed that this variation in the prevalence was due to the different climatic conditions and farming techniques used in the two sampling sites. A similar prevalence rate (8.2%) was estimated by Naz et al. [7] while analyzing the goats from the Lahore district in Punjab. Few reports are available in the literature regarding the prevalence of *T*. *ovis* in small ruminants from the neighboring country India. In recent studies conducted in the Haryana state of India, Nangru et al. reported that 23% of sheep [29] and 43% of goat blood samples [30] were infected with *T*. *ovis*. All these variations in the *T*. *ovis* infection rates could be caused by changes in bioclimatic conditions, geographic regions; tick density; management practices, and biotic factors like gender, age, and host immunity [14].

In another context, the genetic diversity of *T*. *ovis* in sheep has been cited in the literature, but data regarding goats from Pakistan is very limited. Therefore, we analyzed three *T*. *ovis* isolates after sequencing the PCR products amplified from the 18S rRNA gene of *T*. *ovis*. BLAST analysis of the partial sequences confirmed the infection with *T*. *ovis*. In addition, the phylogenetic study revealed that the DNA sequences amplified during this investigation were highly conserved, and they closely resemble the other identified *T*. *ovis* sequences that were isolated from the sheep in Pakistan [10, 14], China [31] and Iran [12], from sheep and goat in Turkey [32, 33] and Burkina Faso [34] and cattle in Tanzania (*unpublished data*) (Fig 2). Our results highlight the need for more detailed investigations from various regions in Pakistan to confirm the genetic diversity of *T*. *ovis* in goats. Finally, it is suggested that more discriminative genes should be selected for the phylogenetic analysis during future studies, and the findings should be correlated with the virulence of this parasite.

Risk factors analysis revealed that *T*. *ovis* prevalence varied according to season, and the highest infection rate was recorded in goats tested in the summer months (Table 1). These observations align with Khan et al. [11], as they had previously reported that the highest *T*. *ovis* prevalence in goats from Bizerte was recorded during the summer. This increase in the prevalence of *T*. *ovis* infection during summer could probably be due to the environmental conditions, like increased relative humidity and temperature that are favorable for the growth and reproduction of ticks and hence result in increased tick exposure to goats [1].

During the present investigation, goats younger than 2 years (young) were more significantly infected with *T*. *ovis* infection than older goats (Table 2). This observation is in line with the finding of Iqbal et al. [15] and Guo et al. [32] as they had also found that younger small ruminants were more likely to be infected with *T*. *ovis* than older ones. The increased frequency of theileriosis in younger animals is probably due to a lack of immunity against this parasite [35]. However, our results are contrary to those of Niaz et al. [27], Khan et al. [11] and Zangana and Naqid [36] as they reported a higher incidence of *T*. *ovis* infection in goats aged 2 years or more. These authors suggested that this result could be because older animals are more frequently going out of farms for grazing, increasing their exposure to ticks and hence the incidence of theileriosis. All the enrolled goat breeds in this study were equally susceptible to *T*. *ovis* infection (Table 2). However, Riaz et al. [1] also reported that none of the goat breeds enrolled in their studies was susceptible to *T*. *ovis* infection more than others.

Infections with *Theileria* parasites can lead to a range of changes in the hematological profile of the host. The severity of the changes depends upon the virulence of the strain, animal breed, and immune status of the host animal [37]. Analysis of hematological parameters indicated an elevated white blood cell count in *T*. *ovis*-infected goats (Table 3). These observations align with Al-Fetly [38] and Mohammed et al. [39] who also reported leukocytosis in *T*. *ovis*-infected goats, respectively, from Iraq and Kuwait. These observations agree that an increased number of white blood cells are a part of the defense mechanism and immunological responses against parasites [37].

Biochemical analysis of serum proteins, enzymes, lipids, hormones and metabolites provides information about the organs and tissues in the body as well as the metabolic state of the animal [40]. The analysis of the studied serum parameters indicated a decrease in creatinine and an increase in BUN ratios in *T*. *ovis*-infected goats, which are critical indicators of renal tissue damage (Table 3). In general, a lower serum BUN concentration is a pathological sign associated with chronic hepatic disease, and it is also an indicator that protein levels are low in one’s diet. In contrast, the increased BUN level indicates that either the body is dehydrated or the kidneys have lost their normal function [39]. A study of the rangeland grazed goats showed that the serum creatinine level varies with certain roughages available in wet and dry grazing periods [41]. These observations were also confirmed by Ullah et al. [42] who reported disturbed liver and kidney function markers in Pakistani goats infected with *T*. *ovis*.

## Conclusions

We report the first evidence of *T*. *ovis* infection in goats in Muzaffar Garh District in Pakistan. *T*. *ovis* was more frequent in goats less than two years of age, and more goats were found infected during the summer and winter. *T*. *ovis* infection disturbed the white blood cell count, serum creatinine, and BUN levels of infected goats.
